# Measuring aviator workload using EEG: an individualized approach to workload manipulation

**DOI:** 10.3389/fnrgo.2024.1397586

**Published:** 2024-06-10

**Authors:** Kathryn A. Feltman, Johnathan F. Vogl, Aaron McAtee, Amanda M. Kelley

**Affiliations:** ^1^United States Army Aeromedical Research Laboratory, Fort Novosel, AL, United States; ^2^Goldbelt Inc., Herndon, VA, United States

**Keywords:** workload, aviation, individualized, electroencephalograph, cognitive state, operator state monitoring

## Abstract

**Introduction:**

Measuring an operator's physiological state and using that data to predict future performance decrements has been an ongoing goal in many areas of transportation. Regarding Army aviation, the realization of such an endeavor could lead to the development of an adaptive automation system which adapts to the needs of the operator. However, reaching this end state requires the use of experimental scenarios similar to real-life settings in order to induce the state of interest that are able to account for individual differences in experience, exposure, and perception to workload manipulations. In the present study, we used an individualized approach to manipulating workload in order to account for individual differences in response to workload manipulations, while still providing an operationally relevant flight experience.

**Methods:**

Eight Army aviators participated in the study, where they completed two visits to the laboratory. The first visit served the purpose of identifying individual workload thresholds, with the second visit resulting in flights with individualized workload manipulations. EEG data was collected throughout both flights, along with subjective ratings of workload and flight performance.

**Results:**

Both EEG data and workload ratings suggested a high workload. Subjective ratings were higher during the high workload flight compared to the low workload flight (*p* < 0.001). Regarding EEG, frontal alpha (*p* = 0.04) and theta (*p* = 0.01) values were lower and a ratio of beta/(alpha+theta) (*p* = 0.02) were higher in the baseline flight scenario compared to the high workload scenario. Furthermore, the data were compared to that collected in previous studies which used a group-based approach to manipulating workload.

**Discussion:**

The individualized method demonstrated higher effect sizes in both EEG and subjective ratings, suggesting the use of this method may provide a more reliable way of producing high workload in aviators.

## 1 Introduction

Ongoing research within the US military continues to explore the possibility of using physiological measures to identify an aviator's cognitive state and subsequently predict performance. Moreover, this is a popular topic in a variety of everyday settings such as civilian driving (e.g., Meteier et al., 2021). Within the US Army, the experience of high workload during flight is of particular concern for its rotary-wing aviators. Here, we define workload as the combination of task demands and the operator's ability to respond to them (Young et al., 2015). Rotary-wing aviators are prone to experience a high workload during many routine flights as the maneuverability of these airframes allow for flight at much lower altitudes, often near obstacles, as compared to fixed-wing airframes. As such, several studies to-date have focused on manipulating workload during simulated flight while measuring physiological changes from the aviator (Feltman et al., 2020, 2021). The overarching purpose of these studies was to identify which physiological measures can reliably differentiate between a high and low workload condition. From identifying reliable physiological measures for differentiating workload, the possibility of using those measures to predict debilitating workload in real-time might be realized. If real-time prediction is possible, then interventions such as adaptive automation or alerts may be implemented to avoid degraded performance. However, multiple challenges remain in place for the realization of real-time prediction, such as development of sensors that can be reliably used within the flight environment. Another substantial challenge is how workload is manipulated within simulated studies. In order to develop a real-time physiological monitoring system to predict aviator performance, it is critical to manipulate workload in meaningful ways. The development of the algorithms that would be needed to drive adaptive automation and/or alerts need to be based on an experience of workload that is comparable to what might be experienced during an actual operational mission. The ability to do so within a simulated environment is critical because of the monetary costs and potential safety risks associated with conducting such experiments within a real aircraft.

However, manipulating workload within an applied research setting is not straightforward. Many factors can influence how an individual experiences workload. For example, Radüntz (2020) demonstrated that working memory capacity influenced how participants experienced workload. Specifically, Radüntz applied their “Dual Frequency Head Maps” (DFHM) method of objectively measuring workload through electroencephalograph (EEG) to a planning and working memory task. Subsequent analyses revealed that participants with higher working memory capacity experienced less mental workload (measured by the DFHM method) as compared to those with a lower capacity. Similarly, Broadbent et al. (2023) demonstrated a relationship with working memory capacity and performance during a high workload driving scenario. Specifically, they found that low working memory capacity scores were associated with worsened performance during a dual-task driving scenario. Importantly, no relationship between capacity scores and performance was found during the lower workload, single-task scenario. Thus providing further evidence that when experiencing high workload, individual differences such as working memory capacity, may influence not only how workload is experienced but also how performance is managed.

Besides innate skills, such as working memory capacity, aviators vary in terms of experience. This experience can range from total flight time (measured in hours within a specific airframe) to recency of various trainings. For example, within Army aviation, practicing how to handle in-flight emergencies is managed at the operational unit level. This can create differences in recency of trainings, as the frequency of such training can vary, but typically occur around once a month (SV Alcock 2023, personal communication, 21 March). Scarpai et al. (2021) examined aviators' physiological response during autorotation procedures. Autorotation is an emergency flight procedure performed during an engine failure in helicopters. This task is known for producing a very high workload (Alam et al., 2023). Scarpari et al.'s study included instructor pilots, all with over 2,000 h of flight experience. These pilots were further divided into three groups who differed based on total experience in conducting autorotations (highly experienced test pilots each with more than 3,000 landings in full autorotation; test pilots with only a few hundred full autorotations; and operational pilots with no experience in full autorotations). In examining the success rates of the different groups, the authors concluded familiarity with autorotation procedures as opposed to *just* total flight experienced was more relevant in successful task execution.

Experience levels will also vary based on what aviators have experienced in real-life flights. In most aviation studies examining workload, aviators are presented with challenging scenarios. For example, in our previous work we have used degraded visual environments to increase workload (e.g., Feltman et al., 2020). Alternatively other researchers, such as Gorji et al. (2023), have used specific flight tasks (precision approach) as a means of inducing workload. In both cases, the individual aviator will vary on whether they have experienced such situations in real-life flights, as well as how recently those occurred. Although it would be possible to control for such factors through recruitment strategies, this is rarely feasible when working within constrained budgets and timelines while targeting a specialized population (in this case, Army aviators).

Similarly, social pressures in highly specialized operational fields can affect workload assessment methodologies. In high expertise fields, such as aviation, the willingness of an individual to admit a task was too demanding or that they were unable to competently perform the task may vary based on perceived consequences of stating true introspective workload assessments. For example, subjects who are experts in their field may downplay their subjectively experienced workload while performance and physiological metrics indicate higher levels of workload (Widyanti et al., 2013; Hancock and Matthews, 2019). Reduced values of reported subjective workload make it appear as if the task was not difficult relative to their own abilities. These subjective results compared with the objective performance and physiological metrics can lead to difficult to interpret dissociations among measures.

Lastly, the task demand experienced in real-life scenarios is often more dynamic than what is experienced in the more static laboratory tasks often used in the academic literature. Regarding aviation research, this can consist of a participant maintaining a specific flight path, while also managing communications and addressing unanticipated activities that occur during flight, such as in-flight emergencies. These dynamic transitions in task demand have been found to significantly alter performance, subjective, and physiological workload metrics in unexpected ways. That is to say, the workload history of an individual (i.e., recent task demand transitions) can influence currently perceived levels of workload. Bowers et al. (2014) demonstrated a physiological recovery lag of EEG-derived gamma activity recordings across difficult-to-easy demand transitions within the Multi-Attribute Task Battery II (MATB-II) aviation simulation software. The difficult-to-easy transition within gamma activity took about 45 s to become steady at the new demand level compared to the easy-to-difficult transition that took only 10 s. Theta activity demonstrated a rapid change at the onset of the demand transition, regardless of direction. These EEG findings were corroborated by Kim et al. (2018) using the MATB to demonstrate task demand induced transitions in neural information flow as derived from effective connectivity analysis. As such, it is critical that simulated flight scenarios attempt to mimic the dynamic changes in task demands that characterize real-flight missions in order to gain more representative physiological measures.

Altogether, the influence of individual differences in working memory capacity, experience, and dynamic qualities of examined tasks has a critical effect on workload assessment metrics. These influences are not commonly controlled for across studies, making results difficult to interpret within and between experiments. Hancock and Matthews (2019) created a framework of workload study outcomes to describe dissociations between workload measures that occur due to factors such as individual differences. Within their framework, a set of 27 different states occur between the potential outcomes regarding the sensitivity of performance, subjective, and physiological measures to the experimental conditions. Vogl et al. (2022) reviewed workload assessments in the aviation literature and identified how published studies faired among the possible outcome states for studies that utilized multiple workload metrics. Overall, the aviation literature has demonstrated reliable associations between workload measures within flight and simulated environments. However, several studies reported dissociations. Further contributing to the dissociations seen within the literature surrounding simulator studies may be related to the inability to fully re-create the realism of the situation. Participants flying a simulator do not feel the same “life and death” fear that would likely be experienced in an actual aircraft under similar circumstances. As such, the performance and physiological data captured are unlikely to be the same as that which would be experienced in a real-life, real-flight scenario. These factors taken together with the risks of publication bias and what does not get reported leaves unanswered questions regarding the best practices to utilize in creating effective workload-inducing scenarios.

In efforts to realize operator state monitoring, it is critical to create scenarios that will induce the operator state of interest. While some operator states may be straightforward, such as sleep-related fatigue which can be induced by altering sleep schedules or requiring prolonged wakefulness, workload is a more challenging state to re-create. Given that workload is the byproduct of the interaction between an individual and the task/environment, both sides must be considered through appropriate assessment to control the resulting workload experienced. Both the individual differences AND the task being performed need to be properly controlled by the experimenter to increase the likelihood of manipulating the workload desired and to mimic real-life conditions. To overcome some of these limitations related to measuring aviator workload and to increase the ecological validity of our manipulations, we departed from the traditional approach of using identical workload manipulations for all participants, and instead individualized how workload was manipulated. In doing so, we anticipate that the associated physiological data will be more representative of what might be present in similar real-life scenarios. To individualize the manipulations, participants visited the laboratory twice. The first visit served the purpose of ascertaining individual workload thresholds (i.e., when becoming overloaded), while the second visit applied the individualized manipulations during experimental flights. EEG data were collected during the flights. The reported study evaluated two hypotheses:

- Hypothesis 1: operationally relevant flight scenarios using individualized workload manipulations will result in distinguishable differences in physiological response and aviator performance compared to low workload, baseline flights. Specifically, during the flight with individualized high workload manipulations, the following patterns will be seen within the frontal EEG data measurements: (a) theta values will *increase*, (b) alpha values will *decrease*, (c) beta values will *decrease*, and (d) a combined index, the beta-ratio [beta/(alpha + theta)] will *increase*.

- Hypothesis 2: the use of individualized workload manipulations produces more robust (in terms of effect sizes) and consistent findings across participants.

## 2 Methods

The U. S. Army Medical Research and Development Command Office of Research Protections Institutional Review Board reviewed and approved the protocol for this study. Researchers conducted all procedures according to institutional ethical standards. Prior to participation, all participants provided informed written consent. The data reported here are a subset of data from a larger study (Feltman et al., 2021).

### 2.1 Participants

Eight male, Army-rated aviators (seven active duty, one National Guard) with a mean age of 37.25 years (*SD* = 3.33) participated in the study. Participants reported a mean time in their military career of 85.63 months (*SD* = 38.14), or ~7 years. Participants were experienced flying the UH60 Black Hawk aircraft (same airframe as that used in the study), reporting a range of 700–2,900 total flight hours with a mean of 1,434.38 h (*SD* = 683.08) within the UH60 over their careers. Participants were monetarily compensated for their participation.

The Beck Depression Inventory (BDI) and State Trait Anxiety Inventory (STAI) were collected and evaluated to identify any elevated scores. Elevated scores on these measures may impact EEG data. Regarding these two measures, all participants scored below the published cut-off criterion suggesting all were free of depression (BDI, *M* = 0, *SD* = 0) and anxiety symptoms (STAI, State *M* = 29.14, *SD* = 2.19, Trait *M* = 28.57, *SD* = 3.91) (respectively, Beck et al., 1996; Ercan et al., 2015).

### 2.2 Devices and materials

#### 2.2.1 Flight simulator

**Figure 1:**
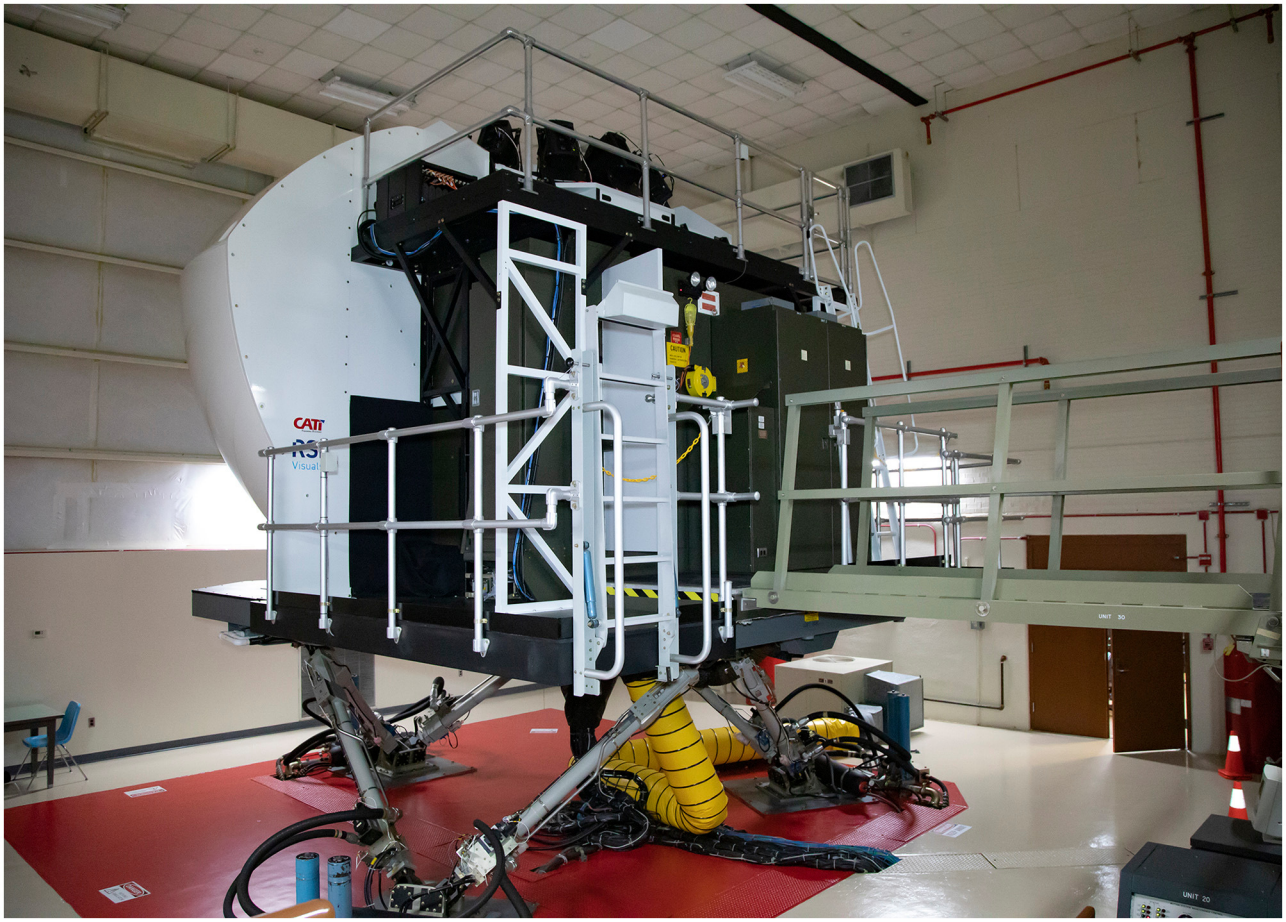
Outside of UH60 full-motion simulator.

**Figure 2:**
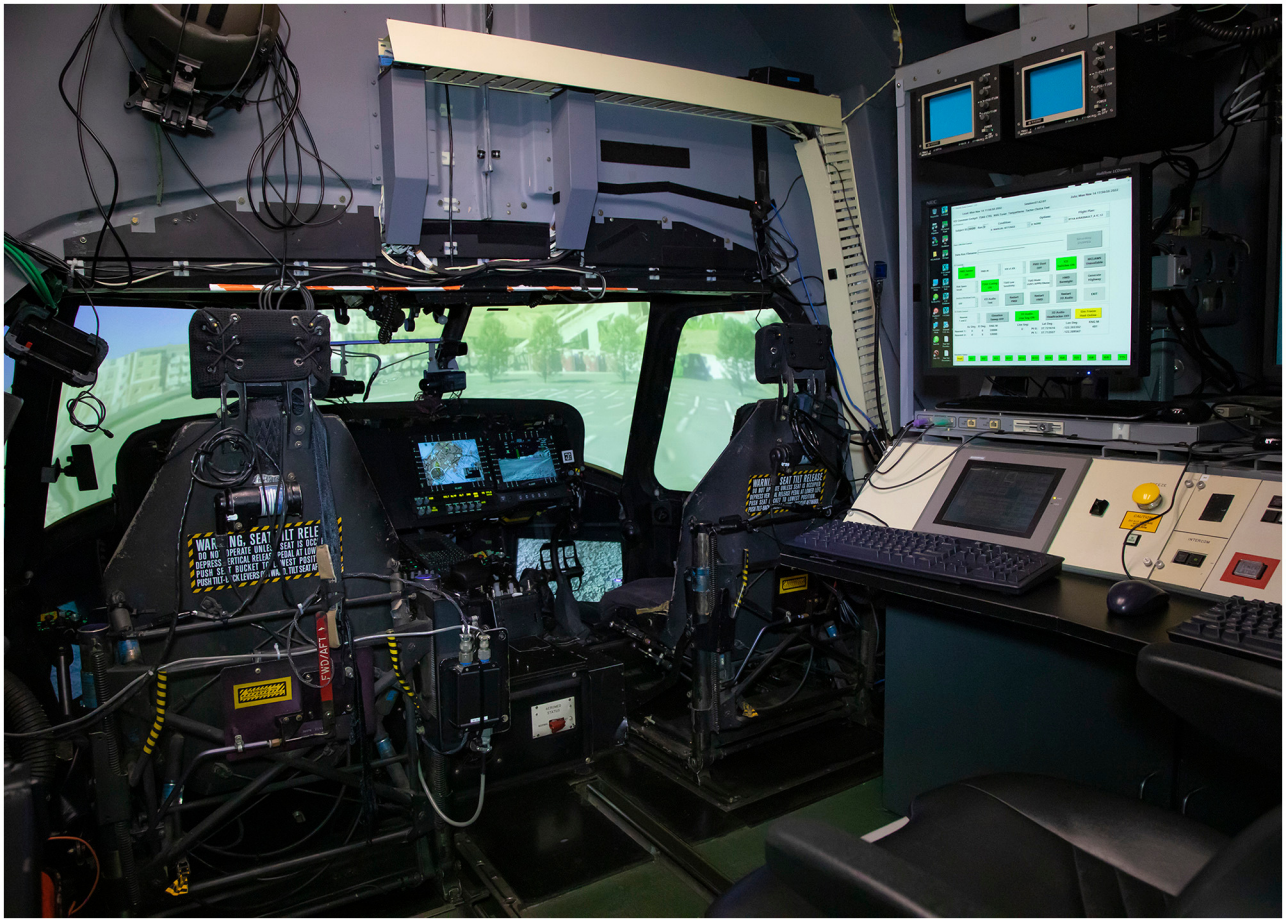
Inside of the UH60 simulator, including control station.

A full-motion UH60 Black Hawk simulator was used (see Figures 1, 2). The simulator is equipped with a six-degree-of-freedom motion system, an instructor/operator station within the simulator, and a separate observer station. Aircraft/simulator state parameter changes were collected at 60 Hz. Settings were manipulated to alter workload by changing environmental factors (e.g., cloud layer, visibility, and turbulence level), and aircraft functionality. Turbulence was scaled from 1 to 7, where 1–3 is considered “light-moderate turbulence,” 3-5 “moderate turbulence,” 5–7 “moderate-extreme turbulence.”

Outcome measures from the simulator included flight variables during the en-route and landing portions of flight. Two variables were measured for performance during en-route, these were: root-mean-squared-deviations of altitude from 200' above ground level and the percent of time airspeed was above 90 knots-indicated-airspeed (KIAS). Two variables measured landing performance, these were: tail velocity at touchdown which provided an indication of speed and direction of the tail of the aircraft at touchdown, and airspeed at touchdown, where the “goal” of velocity is 0 and of airspeed, 0 KIAS.

#### 2.2.2 Subjective measures

Subjective workload was measured using two methods: post-flight and continuously throughout the flight. Workload was measured post-flight using the NASA Task Load Index (NASA-TLX) (Hart and Staveland, 1988). The NASA-TLX required participants to rate workload using a 100-point scale on the following categories: mental demand, physical demand, temporal demand, performance, effort, and frustration. Participants then completed pairwise comparisons of the subscales, which were used for computing a weighted total score, which was the primary outcome measure evaluated. Workload was also rated continuously throughout the flight using the Instantaneous Self-Assessment of Workload (ISAW) (Brennan, 1992; Jordan, 1992). Every 1 min during the simulated flights, participants were prompted through an audio tone to rate workload. Participants then rated workload verbally using a scale of 1 (underutilized) to 5 (excessive). Ratings were recorded in a spreadsheet by a member of the research team. If participants did not rate workload within 5 s of the tone playing, the tone replayed. The tone replayed three times total (each 5 s apart) to prompt a response. Instances when participants did not respond to the prompts were considered a missed response. The next rating prompt occurred 1 min after the initial tone.

The BDI-II (Beck et al., 1996) and STAI (Ercan et al., 2015) were used to provide screening for pre-existing mental health conditions. The BDI-II consists of 21 multiple-choice items that capture affect, cognitions and physical symptoms of depression over the previous 2 weeks. The STAI consists of 40 items that participants rate on a 4-point Likert-type scale. These items capture two types of anxiety: state, or event-dependent anxiety, and trait, persistent demonstrations of anxiety as a personal characteristic. Demographic information, such as age and flight experience, were collected using an in-house developed questionnaire.

#### 2.2.3 Electroencephalograph

The Advanced Brain Monitoring B-Alert X24 wireless wet electrode system recorded EEG activity. The X24 incorporates 20 channels corresponding to scalp locations according to the International 10–20 system. After applying the EEG system, participants' baseline EEG data were collected. This required participants to complete three tasks provided by the B-Alert system. The purpose of these tasks is to create B-Alert's proprietary cognitive state metrics. These metrics, however, were not analyzed in the current study. Rather, we used the B-Alert Live Software (B-Alert Live, 2009) to process all EEG data and provide the outcome variables of interest. This software computes power spectral density (PSD) values by first identifying and removing artifacts, and then performing Fast Fourier Transform (FFT) on the data to calculate the amplitudes of the sinusoidal components for designated frequency bins. These procedures are described in (Berka et al., 2007; B-Alert Live, 2009). Outcome measures for this study included theta (4–8 Hz), alpha (9–13 Hz), and beta (14–30 Hz) PSD values from four frontal channels (F7, F3, F4, F8). Additionally, a combination of the three were used to create the beta-ratio [beta/(alpha + theta)] (Freeman et al., 2000). Note, the beta-ratio was calculated using only data from the frontal channels, whereas Freeman et al. (2000) used a montage that included central and parietal channels.

### 2.3 Procedures

Data collection for this study occurred during the COVID-19 pandemic (November 2020–May 2021). The research team took additional precautions and procedures to ensure the health and wellbeing of participants. All participants were screened for any COVID-19 symptoms prior to entering the laboratory. Participants were required to wear cloth masks throughout the duration of the study procedures. When inside the simulator, members of the research team, including the research pilot (RP), wore N-95 masks. There were no noted issues with communication while wearing masks, as supported by Cave et al.'s (2021) evaluation of mask wearing within the aircraft. Of note, due to the timing of when data collection occurred, the data collection efforts were truncated. The target sample size was 16 participants, but we were required to discontinue at eight participants.

Participants for this study were scheduled for two visits. During the first visit, procedures were completed to determine individualized workload settings. The methods used to determine individualized workload settings have been previously published (Feltman et al., 2022) and are described in the Supplementary material.

#### 2.3.1 Visit one

During the first visit, participants completed consenting procedures. Next participants completed the questionnaires (demographics, BDI, and STAI). After completing the questionnaires, participants were affixed with the EEG and other physiological devices to complete baseline measures.

Following this, participants were trained how to complete the ISAW ratings during the experimental flights and familiarized with the tone prompting when to rate. The rating scale was also posted within the simulator for participants to reference during the simulated flights. Next, participants were escorted by a RP to the simulator to familiarize with its layout and functionality. During this time, the RP verified the participant was able to meet performance criteria for the study.

Next, participants completed four flight scenarios designed to assess their performance and subjective experience to various workload manipulations. Two of the flight scenarios were designed to maintain a low, manageable workload, while two flight scenarios were designed to elicit the experience of a high workload by introducing various workload manipulations throughout the flights. The flight scenarios are described in Section 2.3.3 below. These latter flights introduced manipulations in a linear fashion, whereby the added manipulations continually increased workload. During these flights, participants' performance was continually measured and ISAW ratings were prompted at 1-min intervals throughout. Two RPs monitored the progress of the flight to assess performance, as well as either the Principal Investigator (PI) and/or an Associate Investigator (AI) on the project. One RP was located inside the cockpit acting as co-pilot, while the second RP was located outside the cockpit in a control room. The RP in the control room could view and hear the progression of the flight and acted as air traffic control.

Following completion of these four flights, participants' ISAW ratings were reviewed alongside the observations of the RPs and PI and/or AI. This information was used to determine where participants experienced the greatest amount of workload, and subsequently used to develop the individualized flights for Visit Two (for details on this process, please see Feltman et al., 2022).

#### 2.3.2 Visit two

When participants arrived for the second visit, they were affixed with the physiological devices and baseline data were recorded. Next, participants were briefed by a RP of the upcoming flight scenario. Participants were presented with a scenario where they were recently hired as a tour guide for a San Francisco helicopter tour company. They were informed they would perform two flights. The first flight would consist of a training flight to learn the tour route. This flight did not include any workload manipulations (beyond those that naturally occur during flight, such as performing takeoff procedures). The second flight was to be their first flight as a tour guide for the company, with the RP as the first “patron.” During this flight, the individualized workload manipulations were introduced throughout. These scenarios are described in the Supplementary material. The order of flights remained the same for all participants (Flight 1 = training flight; Flight 2 = tour guide flight). EEG data, flight performance, and ISAW ratings (every one min) were collected throughout the flights, with the TLX collected at the end of each flight.

#### 2.3.3 Flight scenario descriptions

##### 2.3.3.1 Visit one flight scenarios

Four flight scenarios were created for the first visit. These consisted of two with minimal workload (“low”) and two with increasing workload (“high”) throughout the scenario. Each scenario lasted ~25–30 min. The scenarios were created in two databases, Alaska and California, such that a low and high workload scenario was constructed within each database location. This allowed us to avoid any confounds potentially due to one database location being more challenging than the other (due to factors such as terrain).

Within all four scenarios, workload manipulations were based on four domains, broadly representative of Wickens' Multiple Resource Model (Wickens, 2008): cognitive, physical, visual, and auditory. Examples of manipulations used within each domain are as follows:

- Cognitive: emergency procedure (e.g., hydraulic pump #2 failure; engine failure).
- Physical: increased turbulence, approach to landing within a confined landing zone.
- Visual: decreased visibility, precipitation added.
- Auditory: increased radio calls.

In using these domains, the research team was able to identify the domains that were most challenging for each individual pilot. This enabled the research team to focus on those specific domains for introducing workload manipulations during the individualized Visit Two flights (described next). Please see the Supplementary material for the description of the process used to individualize the flights.

##### 2.3.3.2 Visit two flight scenarios

Two flight scenarios were created for the second visit. One flight scenario was designed to consist of a low, manageable workload and served as the familiarization flight. This flight always occurred first, as it served to provide the participants with instructions regarding the second flight. The second flight was designed to create the experience of high workload, with the individualized manipulations introduced within the flight.

The participants were briefed that they were newly hired tour-guide pilots for a helicopter tour company based out of San Francisco, CA. The database used for the flights was located within San Francisco. During the first flight, the research pilot acted as the pilot training the “new hire” (participant) on the tour route. The route included typical San Francisco attractions, such as the Golden Gate bridge and the 49ers stadium. These locations were used as visual reporting points. For the second flight, the research pilot acted as the first patron for the “new hire.” This allowed us to require the participant to control the aircraft completely on their own, without assistance from the research pilot. The participants guided the research pilot on the tour route, and the individualized workload manipulations occurred throughout. Table 1 below describes the order of events throughout the flight, with the standard workload manipulations that all participants experienced. In viewing the table, the items in the “Event” column were the same events and the same order for both flights. The items in the “Workload Manipulations” column only occurred during the second flight.

**Table 1:** Visit two flight scenarios.

**Event order**	**Event**	**Workload manipulations**	**Workload domain**	**Estimated time point**
1	Takeoff	Patron begins asking questions	Audio/cognitive	1:00 m
2	En route	ATC requests weather report	Audio/cognitive	3:00 m
3	En route (Giants Stadium) VFR reporting point	Hydraulic pump #2 failure malfunction	Physical/cognitive	5:00 m
4	En route	Radio frequency change	Audio/physical	5:00 m
5	En route (Golden Gate Bridge) VFR reporting point	Precipitation/reduce visibility to 2 SM	Visual/physical	8:00 m
6^*^	En route (Golden Gate Bridge) VFR reporting point	Increase turbulence to level 5	Physical	8:00 m
7	En route (Golden Gate Bridge) VFR reporting point	Left pedal drive forward malfunction	Physical/cognitive	9:00 m
8	En route (Golden Gate Bridge) VFR reporting point	Radio frequency change	Physical/cognitive	9:30 m
9	En route (Golden Gate Bridge) VFR reporting point	Squawk code change	Physical/cognitive	9:30 m
10	En route (49ers Stadium) VFR reporting point	Subject still flying with pedal drive EP	Physical	14:00 m
11	En route (49ers Stadium) VFR reporting point	Reduce visibility to 1SM	Physical/visual	16:00 m
12	En route (49ers Stadium) VFR reporting point	Increased turbulence to 7	Physical	17:00 m
13	En route (49ers Stadium) VFR reporting point	Engine #2 failure malfunction	Physical/cognitive	18:00 m
14	En route (49ers stadium) VFR reporting point	Radio frequency change	Audio	19:00 m
15	En route (polo fields) VFR reporting point/landing zone	None	NA	20:00 m
16	Landing at polo fields	None	Physical	22:00 m

VFR, visual reporting point; ATC, air traffic control; EP, emergency procedure.

^*^#2 Fuel Pressure Low malfunction was introduced here during the baseline workload flight. While workload minimally increased, the research pilot aided in the response to the malfunction.

Regarding the individualized manipulations, Table 2 below indicates what manipulations were introduced and the number of pilots who experienced those additional manipulations. Some of the pilots received multiple manipulations. These manipulations occurred throughout the duration of the flight.

**Table 2:** Visit two individualized workload manipulations.

**Workload domain**	**Description**	**Pilots who received manipulation**
Auditory	Increase frequency of patron chatter throughout flight and increase frequency of radio calls with 40% ownship calls	*n* = 7
Cognitive	Increased tasks such as GPS manipulation, radio frequency changes, and weather reports	*n* = 3
Physical	Start turbulence at 5 and increase to 9	*n* = 2

Visual manipulations were constant across pilots. Additional scenario details can be found in the Supplemental material.

## 3 Results

### 3.1 Statistical approach

Hand-entered data were double-checked for accuracy using a 10% random sample validation check. Any errors found resulted in double-checking all data entry. Prior to analyses, all electronically recorded data were inspected for any impossible values or output errors. Distributions of performance and questionnaires were evaluated for normality and inspected for outliers exceeding three standard deviations from the mean (no outliers were identified). Analyses were completed using R Studio, version 4.0.2 and SPSS version 25, with “effectsize” (Ben-Schachar et al., 2020) used for effect size calculations. As the purpose of Visit 1 was to determine workload manipulations for Visit 2, results of the data collected during that visit are not reported here. Hedge's *g* is reported for effect size as it provides an unbiased effect size adjusted for small samples (Turner and Bernard, 2006).

### 3.2 Hypothesis one results

To evaluate the first hypothesis, the efficacy of workload manipulations was first evaluated. To do so, paired-samples *t*-tests were used to evaluate the subjective workload ratings. ISAW ratings were aggregated across the entirety of the baseline and high workload flight. TLX total-weighted ratings (single score) were used to evaluated TLX ratings. The efficacy of the workload manipulations was supported, with higher ratings during the high workload condition for both subjective measures (see Table 3 below).

**Table 3:** Subjective workload descriptive statistics and *t*-test results.

	**Metric**	**Baseline**		**High workload**		**Paired-samples** ***t*****-tests**		
		**Mean**	**SE**	**Mean**	**SE**	***t* (7)**	***p***	***Hedge's g***
Subjective workload	ISAW ratings	2.03	0.16	3.42	0.19	6.00	< 0.001	1.88
	TLX-weighted rating	19.25	4.42	73.71	6.26	10.04	< 0.001	3.16
En-route flight performance	RMSD altitude	64.7	4.49	70.4	5.67	0.70	0.51	0.22
	Above 90 pct	29.2	8.80	30.6	12.5	0.13	0.90	0.04
Landing flight performance	Tail velocity touchdown	2.46	0.40	5.62	1.20	2.19	0.06	0.69
	Airspeed at touchdown	3.60	0.76	19.08	6.32	2.34	0.05	0.73
EEG	Alpha	0.1592	0.002	0.1548	0.002	−2.46	0.04	0.77
	Beta	0.4051	0.006	0.4121	0.005	2.11	0.07	0.66
	Theta	0.1727	0.007	0.1607	0.005	−3.49	0.01	1.10
	Beta ratio	1.2303	0.0511	1.3112	0.0385	3.07	0.02	0.96

Next, we evaluated whether the high workload condition impacted aviator performance. Paired samples *t*-tests were again used. The performance results did not reach statistical significance (see Table 3 below).

Finally, the EEG metrics were evaluated. Here, frontal alpha, beta, theta, and the beta-ratio were significantly different in the high workload condition compared to the baseline condition (see Table 3 below).

### 3.3 Hypothesis two results

To evaluate whether the individualized method used in this study resulted in a higher experience of subjective workload and associated physiological changes as compared to past studies, the subjective workload ratings and EEG data from this study were evaluated alongside those collected from other similar studies at our laboratory (Feltman et al., 2020; Kelley et al., 2020).

Full descriptions of the studies can be found within the referenced technical reports. Briefly, the Feltman et al. (2020) report consisted of two separate studies. The first study, referenced as Study One, used basic cognitive tasks where workload was manipulated between low and high task demands (some tasks included medium, but those results were not used in these analyses). Various physiological measures were recorded, but only EEG is referenced here. The second study, Study Two, used a variety of flight scenarios where workload was manipulated with low and high task demands. The same physiological measures were collected, with only EEG reported here. The current study is referenced as Study Three. The Kelley et al. (2020) report (Study Four) evaluated workload using an unmanned aerial systems task paradigm. Participants performed a supervision task that required them to visually identify targets. Workload was manipulated in this study by number of targets presented each minute.

To evaluate workload ratings, first the NASA TLX ratings during high workload conditions were compared across the four studies. A linear regression model with repeated measures was used to evaluate the effect of Study number on NASA TLX total ratings. All assumptions were met prior to running this model. The results found that the NASA TLX ratings in the high workload conditions of Studies One, Two, and Four were significantly different from those of Study Three (current study). Workload ratings in Study Three were significantly higher, see Table 4 below for means.

**Table 4:** Subjective workload ratings for all studies.

**Study**	***n***	***Mean***	***SD***
One (Feltman et al., 2020)	58	50.2	22.8
Two (Feltman et al., 2020)	183	53.9	20.4
Three (current)	8	73.7	17.7
Four (Kelley et al., 2020)	39	46.5	16.8

The reported n is the number of observations collected. For example, in Study One, 16 participants completed the study. However, there were multiple instances of workload ratings. Thus, the number of observations is 58.

Next, effect sizes of the EEG metrics from the four studies were compared (see Table 5). Here, once again, the effect sizes [using Cohen's convention as reference: *g* < 0.2 negligible; *g* = 0.2 small; *g* = 0.5 medium; *g* = 0.8 large (National Institute of Standards and Technology, 2018)] from Study 3, the current study, were much higher compared to most of the other three studies. Based on the evaluation of the TLX ratings and EEG effect sizes, it is evident the workload manipulations were most effective in Study Three.

**Table 5:** Study EEG effect sizes.

**EEG measure**	**Study One**	**Study Two**	**Study Three**	**Study Four**
Alpha	0.46	0.10	0.77^*^	0.51^*^
Beta	0.15	0.09	0.66^*^	0.02
Theta	0.48	0.07	1.10^†^	0.25
Beta ratio	0.11	0.005	0.96^†^	0.25

^*^Indicates medium effect size.

^†^Indicate large effect sizes.

**Figure 3:**
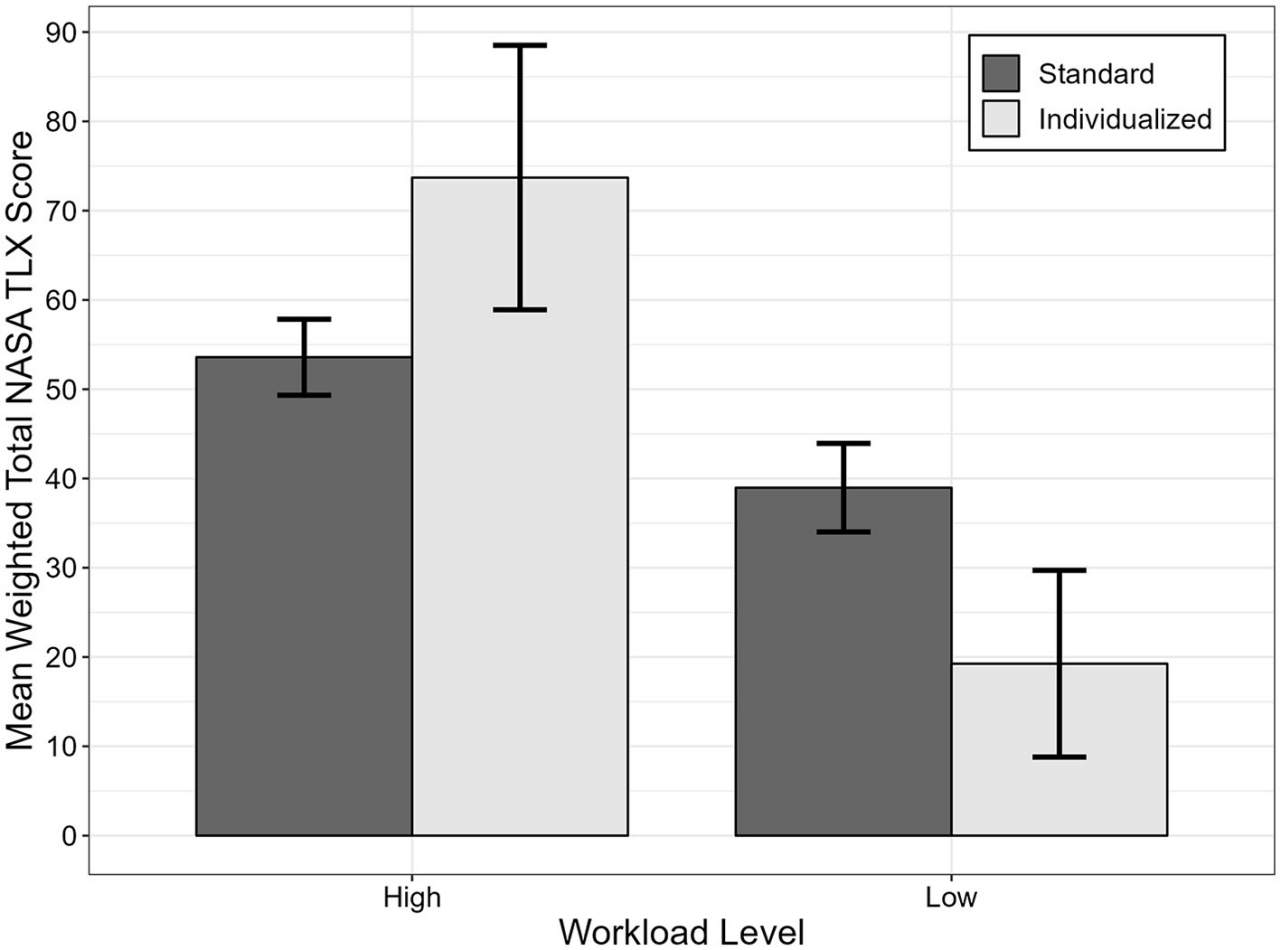
NASA TLX ratings for individualized vs. standard group approach.

In addition to comparing across the four studies, the TLX ratings from the current study were also compared to those from Study 2 using a 2 (workload: high, low) × 2 [study: 2 (standard approach), current (individualized approach)] mixed-model analysis of variance (ANOVA). The purpose of selecting Study 2 for comparison was due to the similarity across the two studies. Both studies manipulated workload within the UH60 simulator and included aviators as participants. This resulted in a significant interaction effect where the difference in ratings between high and low workload conditions was exaggerated with the individualized approach compared to the group approach (see Figure 3 below), *F*_(1, 29)_ = 71.50, *p* < 0.001, partial η^2^ = 0.71.

Further, regarding the individualized approach used in this study, we also identified several consistent findings:

- All eight participants reported higher NASA TLX ratings during the high workload flight, with an average of 54 points higher and a range from 34 to 78 points higher compared to the low workload flight.
- All eight participants reported higher ISAW ratings during the high workload flight, with an average of 1.4 points out of 5 higher and a range from 0.95 to 2.45.
- Seven out of eight participants had lower theta values and higher beta-ratio values during the high workload flight.
- Six out of eight participants had lower alpha values and higher beta values during the high workload flight.
- Seven out of eight participants had a higher airspeed at touchdown during the high workload flight, with average of 15.5 kts higher than the low workload flight.
- Six out of eight participants had a harder landing (higher tail velocity at touchdown) during the high workload flight, with an average of 3.2 kts higher compared to the low workload flight.

Table 6 below shows the difference in measurements (high workload value – low workload value) for each participant.

**Table 6:** Differences in values from high to low workload for current study.

**Participant**	**Alpha**	**Beta**	**Theta**	**Beta ratio**	**NASA TLX**	**ISAW**	**Tail velocity touchdown**	**Airspeed at touchdown**
A	−0.0080	−0.0032	−0.0079	0.0594	64.7	1.41	4.25	5.30
B	−0.0117	0.0113	−0.0209	0.1547	77.7	2.22	7.37	−3.70
C	0.0043	−0.0058	0.0051	−0.0598	50.7	2.45	3.59	7.03
D	−0.0035	0.0044	−0.0084	0.0563	67.0	1.41	−0.11	2.68
E	−0.0078	0.0116	−0.0183	0.1250	55.7	1.32	−1.80	12.38
F	−0.0068	0.0144	−0.0232	0.1301	34.0	0.57	0.93	38.24
G	0.0000	0.0015	−0.0043	0.0267	51.7	0.95	0.74	11.61
H	−0.0023	0.0214	−0.0186	0.1557	34.3	0.81	10.32	50.27

## 4 Discussion

This study sought to evaluate whether an individualized approach to workload manipulations would produce more robust and consistent findings across participants when evaluating performance, subjective and physiological responses in simulated flight. Results of the current study found that the use of individualized workload manipulations during simulated flight produced significant differences in physiological and subjective responses, thus partially supporting the first hypothesis. However, no significant differences in performance were noted. In comparison to our other studies where workload was manipulated at the group level, we found support for the individualized approach producing more robust and consistent findings with the EEG and subjective data, thus supporting the second hypothesis.

Regarding the physiological responses, two (theta values and the beta-ratio) of the four measures reached large effect sizes, while the remaining two (alpha and beta values) reached medium effect size values. This finding is in line with a recent meta-analysis of studies measuring EEG during the evaluation of cognitive workload (Chikhi et al., 2022). Although the review did not consider combinations of these EEG bands, such as the beta-ratio, the review reports significant differences between alpha, beta and theta values in comparison of low and high workload conditions. Moreover, Chikhi et al.'s (2022) review included studies that used a range of scenarios for workload evaluation, including videogame playing and surgical tasks. Limiting consideration to only those studies that included frontal EEG measurements, as that was what was examined in the present study, only four of the 13 studies used realistic tasks [air traffic controller task, Dasari et al., 2017; simulated flight task (MATB), Hsu et al., 2015; surgical task, Morales et al., 2019; and an airline selection multitasking task, Puma et al., 2018]. Additionally, Chikhi's review included experience levels. From this review, only Morales et al. (2019) included participants who had expertise in the task under study (a surgical task). Thus, suggesting that our findings are in line with recent studies using realistic tasks with experienced participants completing a realistic task in their area of expertise. This is important to note given that most studies evaluating workload, even within the aviation literature, using EEG measures have typically included non-realistic tasks or low fidelity simulators (see van Weelden et al., 2022 for a review). Therefore, by demonstrating a similar pattern of findings within our study as that of Morales et al. (2019), who also used a realistic task with experienced participants, is promising.

The lack of significant findings in performance variables between the high workload and baseline flights in the current study is likely related to the small sample size and having pilots who were highly experienced. Thus, we lacked power to detect significant differences in flight performance. However, it is notable that for each of the landing variables (airspeed at touchdown, tail velocity), the majority of participants demonstrated worse performance in the high workload condition (airspeed at touchdown = seven participants; tail velocity = six participants). Moreover, it is noteworthy that the average airspeed at touchdown in the high workload condition was 19.08 KIAS compared to 3.60 KIAS in the low workload condition. Aviators aim for 0 KIAS at touchdown (Feltman, 2023), thus a higher landing speed is suggestive of difficulties during flight. Considering that in the high workload condition, participants were averaging 19.08 KIAS is a significant finding from a practical sense, even though we were unable to detect statistically significant differences between conditions.

Another potential explanation for not seeing statistically significant differences in performance data may be due to the use of individualized workload conditions for each pilot. With the high workload conditions tailored to the pilot, they were sufficiently challenged (as indicated by subjective appraisals of workload), but not overloaded to the point of catastrophic failure in the task. Pilots were able to recruit enough cognitive resources to maintain performance criteria while task-related effort increased to compensate for the additional demand, as indicated by the increase in workload indicated by subjective and physiological metrics. As referenced in the performance-workload relationship in Vogl et al. (2022), dissociation of performance metrics from subjective and physiological metrics are to be expected as workload increases toward the “red line,” a critical point where workload becomes too much to handle, and performance fails. This highlights the criticality of utilizing operator state monitoring to predict workload prior to performance failure. Furthermore, these results provide additional evidence for individualizing the workload experienced by each participant in research settings to ensure that workload is experienced in the same manner relative to natural abilities.

Besides landing performance, we also evaluated deviations in altitude and airspeed during the en-route portion of flight. Given the complexity of the flights, these en-route measures were likely not sensitive enough to identify differences in performance. Specifically, the aviators faced a number of different manipulations during flight, such as in-flight emergency events and increased communications. Examining this data across the entirety of the en-route portion of the flight may have missed subtle performance changes that occurred in conjunction with the introduction of one of the workload manipulations. However, a limitation to our individualized approach was that participants experienced different manipulations in-flight and sometimes at different times. Thus, we could not evaluate performance changes in response to these manipulations equally across participants. Future analyses of these data may look at within-subject responses to workload manipulations to examine the influence of dynamic task demand transitions and workload history on each workload metric. In addition, there may also be more sensitive measures of performance changes that were not evaluated here. More relevant measures may have included control inputs, which has received greater attention in recent studies and may be a better indicator of performance across various situations/scenarios (e.g., Ledegang et al., 2024). However, we had not programmed our simulator to capture the variables needed to compute such measures at the time of the study. This is something we intend to address in future studies.

The findings we do report here, however, move the needle forward in regard to designing a reliable method of inducing workload in a highly skilled population. Given that aviators in general have the skillsets for managing high task loads, it is critical to have a method for inducing a high workload so that we can accurately measure the physiological response. While we were not able to reach statistical significance with performance, we did find that we could get more consistent physiological and subjective responses, which is often missed in the literature. Additionally, it is worth considering that although our performance measures did not reach statistical significance, we did identify a trend toward worse performance and identified what could be considered as practical significance. Much of the literature surrounding workload finds some sort of disassociation in measures (see Hancock and Matthews, 2019 for a review). However, here we were able to report on subjective and physiological measures that complemented each other, and were more successful, based on large effect sizes, than our previous efforts in measuring workload (Feltman et al., 2020; Kelley et al., 2020). Utilizing an individualized, yet controlled approach, such as this, in studies aimed at evaluating aviators' physiological response in-flight, may hold greater promise in recreating similar physiological output as that in real flight. Doing so will enable more precise measurements which can be used in the development of algorithms to predict aviator degradation in real-time, without the need for costly and risky in-flight data collections. Rather, in-flight data collections can be used to validate developments made from simulated studies.

## 5 Limitations

Several limitations should be noted. The most significant limitation is related to sample size. Due to constraints related to COVID-19, we were unable to reach our desired sample size. However, using Hedge's g which controls for small sample sizes, we were still able to identify significant effect sizes. The study should be replicated with a larger sample size with a wider range of experience levels to better determine the utility of this method. The comparison across previous studies was also limited in that different participants were in each study. A more meaningful comparison would include the same participants across studies to better account for individual differences.

## Data availability statement

The datasets presented in this article are not readily available per institutional policy. However, data can be shared by establishing a data sharing agreement (please contact authors). Requests to access the datasets should be directed at: kathryn.a.feltman.civ@health.mil.

## Ethics statement

The studies involving humans were approved by U. S. Army Medical Research and Development Command Office of Research Protections Institutional Review Board. The studies were conducted in accordance with the local legislation and institutional requirements. The participants provided their written informed consent to participate in this study.

## Author contributions

KF: Writing – review & editing, Writing – original draft, Methodology, Funding acquisition, Conceptualization. JV: Writing – review & editing, Writing – original draft. AM: Writing – original draft, Formal analysis, Data curation. AK: Writing – review & editing, Methodology, Conceptualization.
