# Supplementary material for: Measuring aviator workload using EEG: an individualized approach to workload manipulation
Source: Front Neuroergon. 2024 Jun 10;5:1397586. doi: 10.3389/fnrgo.2024.1397586 (PMC11197431; doi:10.3389/fnrgo.2024.1397586)
Supplement: Supplementary file 1 [file Table_1.docx]

Supplementary Material

Measuring Aviator Workload using EEG: An Individualized Approach to Workload Manipulation

Kathryn A. Feltman^1^*, Jon Vogl^1^, Aaron McAtee^1,2^, Amanda M. Kelley^1^

^1^United States Army Aeromedical Research Laboratory, Fort Novosel, AL, United States

^2^Goldbelt Inc., Herndon, VA, United States

*** Correspondence:**Corresponding Author
kathryn.a.feltman.civ@health.mil

# Description of Performance Assessment for Individualizing Scenarios

In order to develop the individualized scenarios, the team of two research pilots (RP) and Associate and/or Principal Investigator observed and reviewed performance and workload ratings from the First Visit’s flights. In order to do so, the following steps were used:

1. The RPs were trained by the Principal Investigator on the four sources of workload domains used. The definitions used are in the table below.

Table 1. Workload Domain Definitions

| - **Workload Domain** | - **Definition** |
| --- | --- |
| - Cognitive | - Understanding information, evaluating situations, and decision making (e.g., flight planning, fuel calculations, and correlating performance parameters) |
| - Auditory | - Obtaining information through hearing (e.g., warning tones, communications from co-pilot or traffic controllers) |
| - Visual | - Obtaining information through vision (e.g., scanning instruments, looking outside the cockpit, reading a map) |
| - Physical | - Physical stress and coordination requirements, including movements (e.g., control inputs to cyclic or collective, pushing buttons or turning knobs) |

*Note*. Definitions were taken from Webb et al. 2010

The RPs used this information, along with their training as Instructor Pilots, to observe how the pilot-participant’s performance changed with the introduction of the different workload manipulations. The RP made notes during the flight to describe these changes. Such notes included, “Participant tended to drop calls,” “Participant was visually distracted and drifted into side of mountain.”

1. The Associate / Principal Investigator reviewed the Instantaneous Self-Assessment of Workload ratings with the RPs. Using these ratings, which were given every 1 min during flight, the RPs and AI/PI evaluated workload ratings given at the approximate timing of the introduction of different workload manipulations.
2. Using the RPs’ observations during flight and an examination of ISAW ratings in conjunction with workload manipulations during flight, the workload domains of greatest difficulty for each participant were identified. This was done by first taking the RPs’ recommendations (e.g., participant struggled with maintaining flight parameters when radio calls increased and dropped some of the calls), and then by examining whether the ISAW ratings were in agreement (e.g., higher ratings during these workload manipulations). If so, the workload domain for which the manipulation occurred in was identified for inclusion in Visit Two’s high workload flight. Additionally, the AI/PI reviewed the ISAW ratings for any elevated ratings that were not identified by the RPs (none were identified).
3. Finally, some participants were identified as requiring multiple sources of workload manipulations. These participants were identified during the above-mentioned process by the RPs. Specifically, the RPs made note of participants who were less impacted by the introduction of the singular workload manipulations. It was then determined that to sufficiently increase their workload, multiple manipulations would be introduced. Table 2 below specifies which manipulations participants received.

Table 2. Individual Workload Manipulations

| **Workload Domain** | **Description** | **Pilots Who Received Manipulation** |
| --- | --- | --- |
| Auditory | - Increase frequency of patron chatter throughout flight and increase frequency of radio calls with 40% ownship calls | A, C, D, E, F, G, H |
| Cognitive | - Increased tasks such as GPS manipulation, radio frequency changes, and weather reports | B, F, G |
| Physical | - Start turbulence at 5 and increase to 9 | B, G |
